# Revealing the Mechanisms of Protein Disorder and N-Glycosylation in CD44-Hyaluronan Binding Using Molecular Simulation

**DOI:** 10.3389/fimmu.2015.00305

**Published:** 2015-06-16

**Authors:** Olgun Guvench

**Affiliations:** ^1^Department of Pharmaceutical Sciences, University of New England College of Pharmacy, Portland, ME, USA

**Keywords:** CD44, hyaluronan, binding, free energy, molecular dynamics, glycosylation, inhibition, unfolding

## Abstract

The extracellular N-terminal hyaluronan binding domain (HABD) of CD44 is a small globular domain that confers hyaluronan (HA) binding functionality to this large transmembrane glycoprotein. When recombinantly expressed by itself, HABD exists as a globular water-soluble protein that retains the capacity to bind HA. This has enabled atomic-resolution structural biology experiments that have revealed the structure of HABD and its binding mode with oligomeric HA. Such experiments have also pointed to an order-to-disorder transition in HABD that is associated with HA binding. However, it had remained unclear how this structural transition was involved in binding since it occurs in a region of HABD distant from the HA-binding site. Furthermore, HABD is known to be N-glycosylated, and such glycosylation can diminish HA binding when the associated N-glycans are capped with sialic acid residues. The intrinsic flexibility of disordered proteins and of N-glycans makes it difficult to apply experimental structural biology approaches to probe the molecular mechanisms of how the order-to-disorder transition and N-glycosylation can modulate HA binding by HABD. We review recent results from molecular dynamics simulations that provide atomic-resolution mechanistic understanding of such modulation to help bridge gaps between existing experimental binding and structural biology data. Findings from these simulations include: Tyr42 may function as a molecular switch that converts the HA-binding site from a low affinity to a high affinity state; in the partially disordered form of HABD, basic amino acids in the C-terminal region can gain sufficient mobility to form direct contacts with bound HA to further stabilize binding; and terminal sialic acids on covalently attached N-glycans can form charge-paired hydrogen bonding interactions with basic amino acids that could otherwise bind to HA, thereby blocking HA binding to glycosylated CD44 HABD.

## Introduction

**Figure 1:**
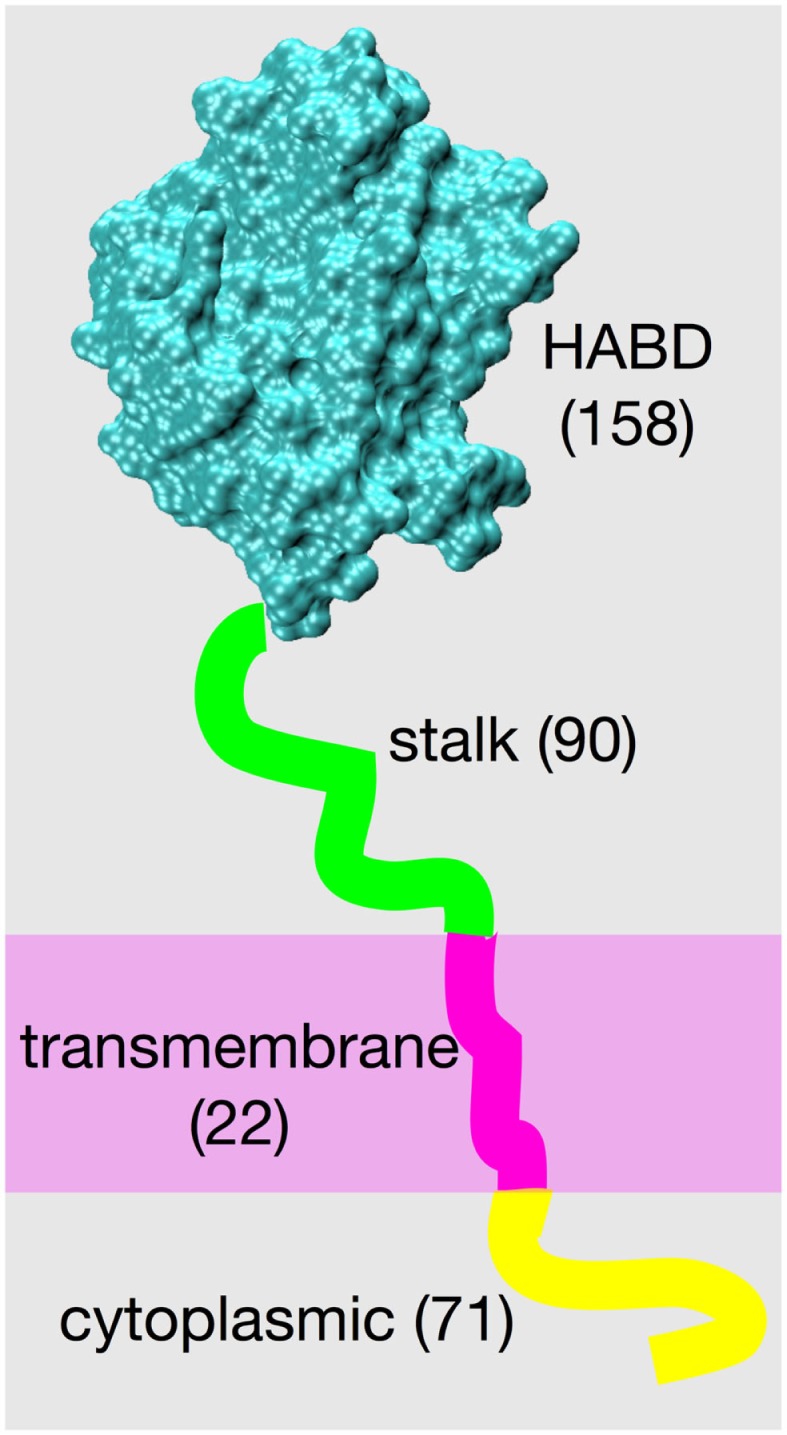
**CD44 structure**. The four different structural/functional regions are denoted by different colors, and labels include the number of amino acids in each region for the standard splice variant “CD44s.” Results of variation in RNA splicing include additional amino acids in the stalk region and loss of amino acids composing the cytoplasmic region. Amino acid numbering for the human isoform begins with residue 21 because of cleavage of a 20-residue N-terminal signal peptide.

The structure of the cell surface protein CD44, from its N-terminus to its C-terminus, consists of a globular hyaluronan binding domain (HABD), a stalk domain, a single-pass transmembrane domain, and a cytoplasmic domain (1, 2). Amino acids located N-terminal to the transmembrane domain are on the extracellular side of the cell membrane, and amino acids located C-terminal to the transmembrane domain are on the intracellular side (Figure 1). Post-translational modifications to CD44 include glycosylation of the extracellular portion (3–5), palmitoylation of amino acids immediately C-terminal to the transmembrane domain (6–9), and phosphorylation of the cytoplasmic domain (10–12). The already-complex structural biology of CD44 is further complicated by variable splicing of the RNA transcript of the *CD44* gene, which yields a variety of different patterns of amino acid insertion into the stalk domain and which modulates CD44 function (1, 13, 14), and by shedding that produces soluble CD44 (15).

Atomic-resolution structures can lead to substantial insight into the function of a biomolecule. Such high-resolution structural information is typically obtained from X-ray crystallography or NMR spectroscopy experiments, with examples being as small as a single zinc finger domain (16) and as large as ribosomes (17) and virus capsids (18). However, not all biomolecules are equally amenable to having their structures solved by these methods. Glycoproteins and proteoglycans are particularly challenging because of the difficulty in obtaining pure samples and the inherent flexibility of these two types of biomolecules. Sample homogeneity is a challenge for glycoproteins and proteoglycans because the carbohydrate component results from non-template-based enzymatic synthesis, leading to carbohydrate microheterogeneity at a given glycosylation site even though the protein component for a given sample is identical throughout (19). The carbohydrate component is also flexible (20, 21), especially in comparison to globular proteins with their well-defined tertiary (three-dimensional) structures, and which therefore were crystallized early in the development of the field of structural biology (22) and still compose the majority of publicly available experimental atomic-resolution structures.

In contrast to globular proteins, which exist in an aqueous environment, transmembrane proteins are located in biological lipid bilayers. Therefore, this environment must be suitably reproduced in samples in order to do experimental structural biology, which can be very challenging (23, 24). Additionally, heterologous expression of transmembrane proteins and subsequent purification can be more difficult than for water-soluble globular proteins because of the limited surface area of the cell membrane for expression, the resulting toxicity to the organism being used for expression, and the subsequent need to reconstitute the protein in a lipid environment after extraction and purification (25, 26).

Intrinsically disordered proteins provide another contrast to globular proteins in that the former lack well-defined unique stable three-dimensional structures (27–29). In X-ray experiments, this results in crystallographic disorder, diffuse scattering, and therefore undefined atomic coordinates (30). While NMR has been used extensively to study intrinsically disordered proteins, solution NMR experiments yield data that represent ensemble averages, which can limit understanding of the various discrete conformations that such proteins may assume (31).

CD44, with its multiple domains, poses a number of challenges for characterization by X-ray crystallography or NMR spectroscopy. The N-terminal HABD, which is similar to globular proteins, is in its biologically relevant form a glycoprotein that has numerous glycosylation sites (1). Furthermore, HABD in the presence of hyaluronan (HA) has characteristics of an intrinsically disordered protein (32, 33). The stalk domain that connects the extracellular HABD to the cell membrane has both N- and O-glycosylation sites (34). And, with alternative splicing, the stalk domain can have proteoglycan characteristics, namely a protein core with glycosaminoglycan (GAG) attachments in the form of chondroitin sulfate and heparan sulfate (35). Recalling that CD44 is a transmembrane protein, in addition to the challenge associated with being located in the cell membrane, the transmembrane domain can be post-translationally modified by the attachment of lipids, which further complicates its structural biology since this modification can alter its interactions with the membrane bilayer (9, 36, 37). Finally, the cytoplasmic domain is likely disordered when not non-covalently bound to intracellular adapter proteins (12).

Atomic-resolution experimental structural biology on CD44 has been largely limited to the ~150 amino acid HABD in its non-glycosylated form because of the many specific challenges above. X-ray structures for HABD exist for both human and mouse isoforms. Human HABD has been reproducibly crystallized in its apo form (i.e., not bound to HA, inhibitors, or peptides) (38, 39), as well as with unidentified peptide (39) found to be non-covalently bound to the face of HABD opposite that of the location of the HA-binding site. However, there are no publicly available X-ray structures of human HABD complexed with either HA or with inhibitors of HA binding. In contrast, mouse HABD has been co-crystallized with both oligomeric HA (oHA) (40, 41) and small-molecule inhibitors (40), as well as in its apo form (41). These co-crystals reveal the binding mode of HA with HABD, which is presumably the same for human HABD given the ~85% sequence identity between the two forms, and the 100% sequence identity of the HA-binding site (42). NMR structures exist for human HABD, both in its apo form (38) and bound to oHA (32). However, unlike X-ray structures with HA, NMR structural characterization of the bound form lacks atomic coordinates for HA and therefore does not provide comprehensive information into the non-covalent atomic contacts between HABD and bound HA. All of these previous structural biology examples are of CD44 HABD; the only non-HABD example of experimental CD44 structure is a complex consisting of a nine amino acid long portion of the 72 amino acid cytoplasmic domain complexed with the radixin FERM domain (12).

Molecular dynamics (MD) is a physics-based approach to the modeling and simulation of biomolecules (43). In all-atom explicit-solvent MD simulations, all the atoms of the system, including those for the solvent, are included as interaction sites for computing the forces in the system. The values of the forces as a function of atomic positions are determined by a combination of a mathematical expression and parameters, commonly called a “force field,” that encodes properties such as the energetic cost to stretch a bond or the energetic benefit of a van der Waals or charge-pairing interaction (44). These forces are numerically integrated to propagate the system, and this is done in an iterative manner to generate a trajectory, analogous to a movie, that shows how the positions of the atoms in the system evolve with the passing of time (45). Typical present-day simulations involve tens of thousands to hundreds of thousands of atoms with trajectory lengths of tens to hundreds of nanoseconds, which require tens to hundreds of millions of consecutive integration steps. MD simulations can be used to determine not only the time-evolution (dynamics) of the system but also the relative probabilities, and therefore free energies, associated with different states (thermodynamics) (46). As such, MD is an especially useful tool for studying flexible biomolecules at an atomic level of resolution, which makes it an ideal complement to experimental structural biology techniques like X-ray crystallography and NMR spectroscopy (47).

Over the past several years, our research group has applied all-atom explicit-solvent MD simulations to extend the understanding of the function of the CD44 HABD. These efforts have aimed to address the following scientific questions: (1) what is the mechanism and associated thermodynamics of a conformational change in an arginine-containing loop at the HABD binding site that is associated with HA binding? (41); (2) why does HABD transition from a well-ordered (folded) three-dimensional structure to one that is partially disordered when it binds to HA? (32, 33, 48); and (3) why do covalently attached sialyated N-glycans inhibit HA binding while unsialyated ones do not? (49–51) This article reviews the contribution MD simulations have made toward developing answers to these questions. We note that others have also recently applied MD simulation to the study of CD44 HABD, with topics including conformational flexibility and the microscopic structure and dynamics of water surrounding HABD (52, 53), and the mechanism and thermodynamics of the ordered to partially disordered transition (54).

## Conformational Switching at the CD44 HABD Binding Site

**Figure 2:**
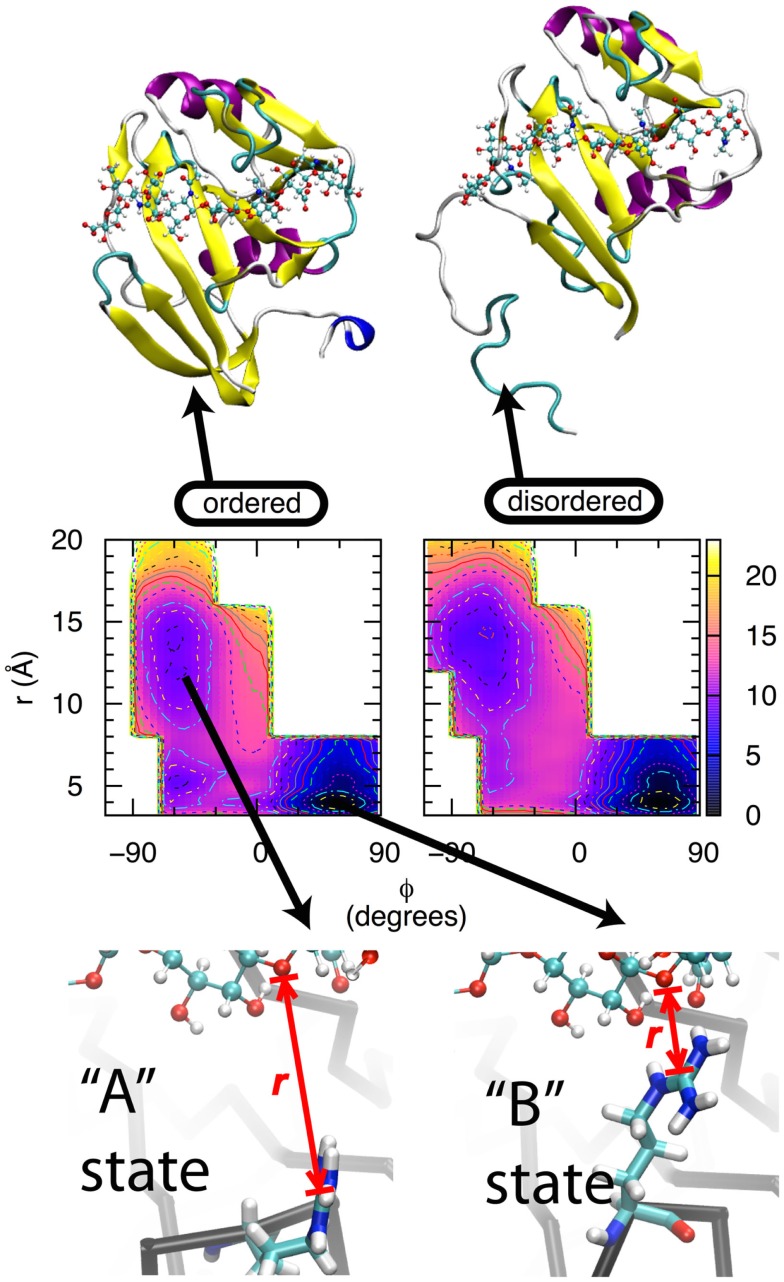
**Thermodynamics of the transition between the A and B states of CD44 HABD**. Bottom row: In the A state, the Arg41 sidechain (tubes) is not in direct contact with oHA (balls-ands-sticks), whereas in the B state, the Arg41-containing loop is in a different conformation that facilitates direct contact. *r* is the distance between the Arg41 sidechain guanidinium central Cζ atom and the ether oxygen atom in the glycosidic linkage connecting GlcNAc3 to GlcUA4 in the bound oHA. Middle row: conformational free energies for the wildtype, ordered (left) and wildtype, partially disordered (right) forms of HABD. Free-energy values are in kcal/mol, contours are every 1 kcal/mol, and data are from Ref. (42). Top row: ordered (left) and partially disordered (right) forms of HABD (ribbons) with bound HA (balls-and-sticks).

Two lines of experimental evidence lead to the hypothesis that direct contact between CD44 Arg 41 and HA is a major source of binding affinity (“Arg41” reflects amino acid numbering in the human form of CD44; the equivalent amino acid is Arg45 is the mouse form. For simplicity, the human numbering will be used throughout this text.). From X-ray crystallography, Arg41 and the loop that contains it change conformation depending upon binding of oHA. In the apo form, the loop is in an open conformation that locates this sidechain too far away to contact oHA if it were present, and HABD is said to be in the “A” state (38, 41). In the complexed form, the loop can be either in the open conformation, or in a closed conformation that facilitates direct contact between the positively charged guanidinium group of the Arg41 sidechain and bound oHA, in which case HABD is in the “B” state (40, 41) (Figure 2, bottom row). From mutation data, the Arg41Ala single point mutation essentially destroys the ability of HABD to associate with oHA (41, 55, 56). This thermodynamic information demonstrates the critical nature of Arg41 in binding. However, the X-ray crystallographic data do not say anything about the thermodynamics of the A and B states, other than they are both sufficiently stable to be trapped as crystals when oHA is bound. And the mutation data do not provide information about the conformation of Arg41 when oHA is bound. We therefore applied MD simulations in an attempt to tie these two previous findings together.

All-atom explicit-solvent MD simulations can be used to determine true thermodynamic free energies for a variety of biomolecular processes (46, 57–60). This is because of two reasons: (1) a model system under study includes water molecules and allows for full conformational flexibility of the included biomolecules, which means both solvent effects and entropic effects are explicitly included (that is, not as an approximation, but as part of the system under investigation); and (2) there exist exact mathematical expressions to determine thermodynamic quantities directly from simulation data. One approach to determining the free-energy difference between two conformational states *x* and *y* of a biomolecule from MD simulation is to compute the reversible work required to transition the system from state *x* to state *y* by integrating the measured average force along the transition path (61). Since free energy is a thermodynamic state function, it does not depend on the actual path used to convert the system from state *x* to state *y*. However, in practice obtaining good numerical convergence as well as plausible biological insight both depend upon determining a physically reasonable transition path.

For CD44 HABD, the two states are A, having an open loop and the Arg41 sidechain separated from bound oHA, and B, with a closed loop and the Arg41 sidechain in direct contact with oHA. While a simple distance between Arg41 and oHA can be used to discriminate between different conformations of the sidechain, it is not immediately obvious that a similarly simple metric can be used to discriminate between the open vs. closed loop conformations. In an effort to identify such a metric, we first compared the conformations of the backbone dihedral angles **φ**, **ψ** for Arg41 and three amino acids on either side: -Lys-Asn-Gly-Arg41-Tyr-Ser-Ile-. This revealed a difference in the Tyr42 **φ** dihedral where in the A state **φ** = −60° and in the B state **φ** = +60° (62). This was followed by MD simulation where force was applied to this dihedral angle to gradually convert it from one value of **φ** to the other. In one case, starting from the A state and increasing **φ** converted the system to the B state, not just with respect to the Tyr42 backbone but also with respect to the separation distance between Arg41 and oHA. In the other case, starting from the B state and decreasing **φ** converted the system to the A state, including breaking of the contact between Arg41 and oHA. In neither case was any force applied to directly affect the Arg41 to oHA distance; rather, changes in this distance spontaneously resulted from changes in the loop backbone conformation. Furthermore, a similar effect could not be achieved using the backbones of other amino acids in the loop (62). Thus, the reaction path for interconversion between the A and B states was defined in terms of two progress variables: the value of the Tyr42 backbone dihedral angle **φ**, and the distance *r* between the Arg41 sidechain guanidinium central Cζ atom and the ether oxygen atom in the glycosidic linkage connecting GlcNAc3 to GlcUA4 in the bound oHA.

Extensive simulations were subsequently done to compute the free energy of the system as a function of the progress variables **φ** and *r* (42). All simulations were of the human HABD (hHABD) complexed with [-4GlcUAβ1-3GlcNAcβ1-]_4_ (“HA8”), which was computationally constructed by combining information from mouse and human structures. The four hHABD-HA8 systems considered were: wildtype, ordered; wildtype, partially disordered; Arg41Ala, ordered; and Arg41Ala, partially disordered. Figure 2, middle row shows the data for both wildtype systems, and the major free-energy minima, which are the most stable states for each system, correspond to A and B.

Wildtype simulation data demonstrate that the B state of the hHABD-HA8 complex is more stable than the A state by ~8–9 kcal/mol. This is true for both the ordered and partially disordered forms of hHABD (Figure 2, top row). Additional simulation data demonstrate that the analogous transition for the Arg41Ala mutant is substantially less favorable at ~6 kcal/mol. From these data, it is possible to calculate the loss in binding affinity associated with the point mutation, with values of 2.2 kcal/mol for the ordered form and 2.3 kcal/mol for the partially disordered form (42). These simulation data are in close agreement with existing experimental data measuring the loss in binding affinity to be 2.5 kcal/mol (41), which helps validate both the force field and the convergence of the simulations. Taken together, these results support the idea that formation of direct contact between HA and the Arg41 sidechain is a substantial source of favorable binding free energy (41).

In contrast to some of these findings, Plazinski and Knys-Dzieciuch, in their simulation studies, found that the **φ**-related free-energy barrier was not correlated with the Arg41-HA distance (54, 63). The authors also observed a low free-energy barrier associated with separation of Arg41 from HA, with the A and B states reducing to an average dynamic structure (54, 63). They suggested that a possible explanation might be differences in the force fields used in their studies (54), since the previous analogous work found the A and B states to be distinct (62). Differences in force fields, which are the underlying physical models used to represent the bonded and non-bonded interactions in such simulations, can indeed cause such differing results. When such differences are inferred based on differing simulation results and conclusions, one possibility is to review the methodology involved in the force field development (44, 64). Another is to compare the outcomes of the particular simulations with the existing experimental data for inconsistencies. In this particular case, relevant experimental data include the crystal structures of the A and B states of HABD complexed with oHA, and the mutation data showing loss of binding affinity in the Arg41Ala mutant. Dynamic averaging would manifest as crystallographic disorder with poorly resolved electron density for Arg41, which is in contrast to the existing crystallographic data. Additionally, rapid equilibrium between short and long separation distances between Arg41 and oHA suggests a weak interaction between them, which is in contrast to the mutation data.

## Binding and Unfolding in CD44 HABD

Early experimental studies of the Arg41Ala mutation that predated the CD44 structural studies also probed the contribution of other basic amino acids by both point and truncation mutations (55). This was a logical course of action in the absence of HABD three-dimensional structure since HA contains a negatively charged carboxylate group every other monosaccharide and Arg and Lys sidechains are positively charged, suggesting the possibility of charge-charge interaction as a mechanism of binding. From that perspective, it is not surprising that these mutations all decrease the strength of binding of HA oligomers. But, taken in the context of the subsequent structural information, the explanation is less obvious, since these additional amino acids, in contrast to Arg41, are located spatially far from the now-known binding site (41). Further complicating the situation is the observation that the portion of HABD that contains these amino acids 153-169 goes from having well-defined three-dimensional structure to becoming unfolded in conjunction with HA binding (32, 33, 48). That is, the change in the conformation, which correlates with the binding of HA, in this span of amino acids located in the C-terminal most region of HABD is what defines the ordered-to-partially disordered HABD transition.

The above suggests two questions: why do basic amino acids distant from the binding site affect affinity? And why is the affinity greater when the sequence containing these amino acids unfolds and becomes disordered? To answer these two questions, we return to the previous set of free-energy data from MD simulations. For wildtype hHABD, the free-energy data associated with the interconversion between the A and B states are qualitatively the same regardless of whether hHABD is in the ordered or partially disordered form (Figure 2, middle row). The same is also the case for the Arg41Ala mutant, where the free-energy change in going from A to B is independent of whether those distant amino acids are folded or not (42) (data not shown here). Not only are there strong qualitative similarities in free-energy data between the ordered and partially disordered forms, but the quantitative values are also very similar. For the A→B transition of the binding site in the wildtype, ordered form, the associated free-energy change is −8.7 kcal/mol, and for this transition in the wildtype, partially disordered form the value is a very similar −7.8 kcal/mol (42). Likewise for the Arg41Ala mutant, in the ordered form the value is −6.5 kcal/mol and in the partially disordered form it is −5.5 kcal/mol (42). The small difference of ~1 kcal/mol is within the precision that can be expected from these particular computational experiments.

In an allosteric mechanism, a conformational change distant from the site affects the energetics at the binding site. In the case of HABD, the independence of the energetics of the binding site A→B transition from the ordered vs. partially disordered form of HABD contradicts the hypothesis that allostery is at work. That is, conformational switching at the binding site appears no more or less favorable if the HABD C-terminal region is folded or unfolded.

Analysis of the MD trajectories that were generated as part of the free-energy experiments yielded a result that, in hindsight, is obvious: flexibility from partial unfolding permits favorable electrostatic interactions between HA and the C-terminal HABD amino acids that cannot occur when the domain is fully ordered. In the ordered form of HABD, the amino acids in question are locked into a folded conformation that keeps them far from bound oHA, while in the partially disordered form, this is no longer the case (Figure 2, top row). Because this span of amino acids is no longer in consistent contact with the rest of the HABD domain, it assumes the properties of a random coil peptide, which through random fluctuations can collide with bound oHA. If this collision happens in a way that brings one of the basic sidechains into close proximity with oHA, a favorable contact can be formed. Unlike the Arg41 interaction, which has a well-defined mechanism based on specific interactions of the sidechain with a particular limited section of bound oHA, the flexibility of the disordered amino acids and the repeating nature of the HA polymer permit the possibility of a wide range of basic sidechain interactions with bound HA (42), consistent with the long-standing finding that a 13-amino acid CD44 peptide spanning Arg150–Arg162 itself will bind HA (65). This wide range may conceivably include interaction with HA bound to an adjacent molecule of CD44, though the present simulations had a single copy of the complex and therefore could not directly address the possibility of such *trans* association.

The above computational experiments do suggest a plausible molecular mechanism by which the ordered to partially disordered transition confers increased binding affinity to oHA. However, the simulations involved either the ordered or the partially disordered form of CD44 hHABD, and therefore do not provide any insight into the mechanism of the ordered to partially disordered transition itself. Independent work has been done toward this end, and with the additional aims of estimating the free-energy profile of the transition and clarifying the role of select amino acids in the transition (54). Connected with the proposed transition mechanism was a free-energy change of +25 kcal/mol, implying the partially disordered form in the absence of HA is very unstable relative to the ordered form. While the sign of the free-energy change, associated with loss of a single beta strand at the edge of a beta sheet, agrees with experiment (32), the magnitude is substantially larger than the folding free-energy values for entire small single-domain proteins, which are typically <10 kcal/mol, including for proteins consisting exclusively of beta strands (66). A further difficulty is that the simulation free-energy data for the analogous transition in the Tyr161Ala mutant are identical to those for the wild type. This is in contrast to experimental data, where the Tyr161Ala mutant constitutively exhibits the partially disordered conformation (33, 48). One possible explanation for these apparent inconsistencies is that the study represents the initial steps of the transition (54), such that extending simulations further along the selected reaction coordinate may result in subsequent decreases in free energy. Another possibility is lack of convergence of simulations given the large scale of the transition (54). This was the case for the free energy for the A→B transition, which involves a much smaller conformational change than the ordered to partially disordered transition. The first study suggested that the A and the B states were essentially equally stable (62), while subsequent work that extended the timescale of the simulations by 40-fold showed the B state to be substantially more stable than the A (42). Importantly, in the case of the ordered to partially disordered transition, the partially disordered form is not a single, well-defined conformation. Rather, the disordered C-terminal HABD amino acids are free to take on a multitude of conformations. Therefore the partially disordered form is actually an ensemble of diverse conformations, and this further complicates computational experiments toward understanding the transition mechanism.

## Inhibition by Glycosylation

N-glycosylation of CD44 HABD is known to have variable effects on CD44 function depending on the nature of the N-glycans. One effect is to block HABD binding to HA (49–51). Another is to make CD44 itself a ligand that binds to lectins (67, 68). The biochemistry behind both of these contrasting functions is related and is modulated by N-acetylneuraminic acid (Neu5Ac) monosaccharide, which is commonly called “sialic acid.” In one case, HABD N-glycans are capped with sialic acids, and this both blocks HA binding and makes CD44 a selectin ligand. In the other case, sialidase activity removes these terminal sialic acids, leaving the bulk of the N-glycan structures intact, and this change restores both HA-binding and removes selectin ligand functionality. While sialidase treatment removes only the terminal monosaccharide from the attached N-glycan, the functional result is the same as removal of the entire N-glycan. For example, Asn point mutation precludes N-glycan attachment and heterologous expression yields non-glycosylated protein. In both of these cases, the functional outcome is the same as sialidase treatment, which implies that inhibition of HA-binding cannot be explained as a consequence of steric blockage of the HABD binding site, since the de-sialylated N-glycan has nearly the same bulk as the sialo-glycan. This mechanism of regulation is not unique to CD44, as the related hyaladherin LYVE-1 demonstrates similar behavior (69).

**Figure 3:**
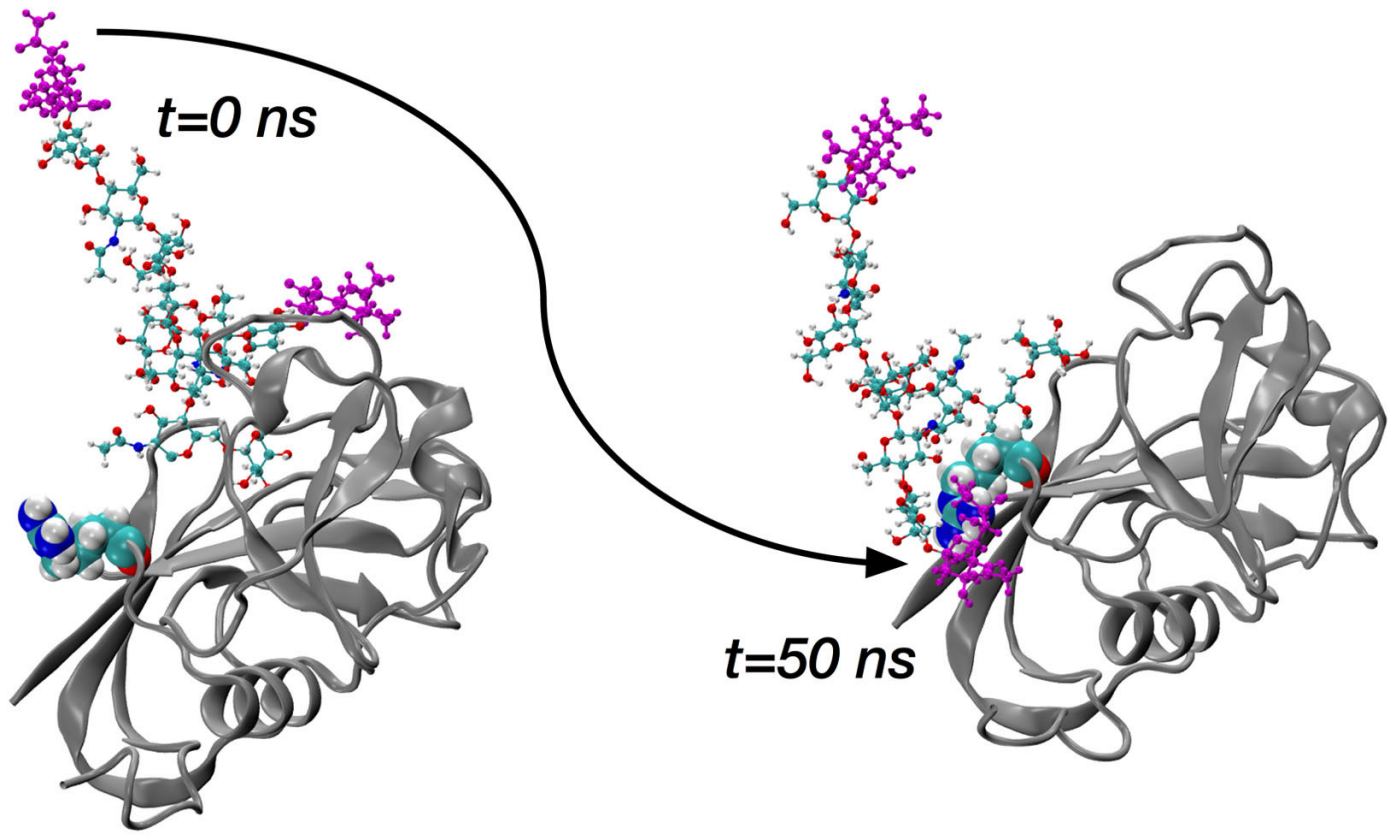
**Molecular dynamics snapshots demonstrating ordered HABD (ribbons) residue Arg41 (van der Waals spheres) forming a charge-paired hydrogen bonding interaction with complex-type N-glycan (balls-and-sticks; sialic acid atoms in purple) attached to Asn25**. Data are timepoints from a single 100-nanosecond (ns) trajectory from Ref. (70).

The sialidase data immediately suggest two sets of simulations of glycosylated HABD: one set with sialylated N-glycan and a second set with asialo N-glycan. Asn25 and Asn120 were selected for computational N-glycosylation based on previous mutagenesis studies showing that cells expressing Asn25Ser and Asn120Ser mutants constitutively bind HA (50). Complex-type N-glycans that were selected (70) based on the existing finding that blocking the metabolic pathway for processing complex N-glycans restores HA binding (71, 72). In conjunction with the two different glycosylation sites and the sialo and asialo forms of the N-glycan, both the ordered and partially disordered forms of CD44 HABD were studied in the simulations. A representative starting conformation of ordered HABD with a sialo glycan attached to Asn25 is shown in the left frame of Figure 3; the analogous asialo form would be missing only the atoms colored purple.

The key finding from the comprehensive set of simulations covering the ordered and disordered HABD paired with sialo and asialo N-glycans is that only in the sialo form do stable, long-lasting non-covalent contacts form between the protein and glycan components. Furthermore, these contacts involve the positively charged sidechains of HABD amino acids and the negatively charged carboxylate groups of the terminal sialic acids (Figure 3). In contrast, asialo glycans form only brief contacts, which is understandable since they lack the negative charge of the sialo form. Long-lasting contacts in the sialoglycan simulations involve Arg41 and Arg154, and these contacts form spontaneously during the simulation and can last for 40–50% of the simulation length (70). Both of these amino acids can directly associate with HA8 when it is bound, based on findings from the computational experiments on partially disordered HABD, as summarized in the previous section. However, their binding with sialic acid is an interaction that would directly compete with their binding with HA8. Therefore, the view that emerges is that, in the sialo form, the covalently attached N-glycans will form charge-paired hydrogen bonding interactions with Arg sidechains known to be important for HA binding. As further evidence, free-energy simulations, similar to the ones for Arg41–HA8 association described in the previous section, demonstrate that the Arg–sialic acid association is indeed thermodynamically favorable by ~1 kcal/mol (70). We do note that these simulations were limited to only the CD44 HABD, which is present in all splice variants of CD44, and that the simulations did not include HA. Clearly, additional work needs to be done to understand the molecular mechanisms by which glycosylation alters binding, since it has been shown that N-glycosylation of CD44 can also facilitate HA binding (73).

## Conclusion

A subset of the computational experiments above suggests the following four conclusions. The first is that the Tyr42 backbone dihedral angle **φ** can act as a molecular switch to convert the HABD HA-binding site from the open A state to the closed B state, which includes the formation of direct contact between HA and the Arg41 sidechain (62). The second is that the B state is more thermodynamically stable, and this stability is due to direct Arg41-HA contact (42). The third is that basic amino acids located distant from the HA-binding site in the ordered form of HABD gain sufficient mobility in the partially disordered form to be able to form direct contacts with oHA and further stabilize binding (42). And the fourth is that terminal sialic acids on covalently attached N-glycans can form charge-paired hydrogen bonding interactions with basic amino acids that could otherwise bind to HA, thereby blocking HA binding to glycosylated CD44 HABD (70). In addition to contributing to the mechanistic understanding of CD44-HA binding, these conclusions may be of utility in the future development of small-molecule modulators of CD44 function (40), especially given the potential for CD44 as a therapeutic target (74–76).

However, it should be kept in mind that the role of Tyr42 as a molecular switch, and the discrete nature of the A and B states of the Arg41-containing loop is contradicted by other computational work (54, 63) Furthermore, there do exist experimental data that are in apparent conflict with the above conclusions. As mentioned previously, N-glycosylation of CD44 can facilitate HA binding (73). And, mutation of positively charged amino acids in the disordered region has been found to enhance HA-binding affinity in the context of both purified HABD and cell surface CD44 (41). Given these differences, further investigation is warranted to achieve a comprehensive consistent view. Finally, while outside the scope of this review, there have been substantial efforts using MD simulations to understand the importance of water molecules and of biomolecular conformational entropy changes in HABD binding with HA (52, 53). Findings from these simulations that can inform development of small-molecule modulators of CD44 function include reduced translational and rotational freedom of water molecules in contact with HABD and HA, and loss of HA flexibility associated with binding to HABD.

The concept of doing computational experiments to address biological questions is appealing, but the technique used here, namely all-atom explicit-solvent MD simulations, requires significant resources. The most obvious resource is computing capacity, since the computing demands are quite large. It is not uncommon for a set of simulations to utilize the equivalent of hundreds to thousands of personal computers running at full speed around the clock for weeks at a time. In practice, this type of computing power tends to be restricted to nationally funded supercomputing centers (77). A second is the development of software capable of making optimal use of modern supercomputers (78–81). And a third is the development of accurate models (i.e., force fields) for the types of molecules that make up the biological systems under study (44, 64, 82). For example, the development of just the carbohydrate component of the force field used in our studies of CD44 HABD involved a collaborative effort spanning over half a decade (83–91). Fortunately, the technique continues to mature, resulting in an increasingly reliable analytical scientific methodology capable of providing accurate and direct insight into questions that could be addressed only indirectly and with great technical difficulty using other approaches.

## Conflict of Interest Statement

The author declares that the research was conducted in the absence of any commercial or financial relationships that could be construed as a potential conflict of interest.
